# Supplementary material for: The Management of Obstructive Sleep Apnea Patients during the COVID-19 Pandemic as a Public Health Problem—Interactions with Sleep Efficacy and Mental Health
Source: Int J Environ Res Public Health. 2023 Feb 28;20(5):4313. doi: 10.3390/ijerph20054313 (PMC10002103; doi:10.3390/ijerph20054313)
Supplement: Supplementary file 1 [file ijerph-20-04313-s001.zip › ijerph-2092777-supplementary.pdf]

International Journal of
Environmental Research and Public Health

MDPI

Table S1. Perceived Stress Questionnaire

Question	1 Almost	2 Sometimes	3 Often	4 Usually
You feel rested	1	2	3	4
You feel that many demands are being made on you	1	2	3	4
you are heritable or grouchy	1	2	3	4
You have many things to do	1	2	3	4
You feel lonely or isolated	1	2	3	4
You find yourself in situations of conflict	1	2	3	4
You feel you're doing things you really like	1	2	3	4
You feel tired	1	2	3	4
You fear you may not manage to attain your goals	1	2	3	4
You feel calm	1	2	3	4
You have too many decisions to make	1	2	3	4
Yyou feel frustrated	1	2	3	4
You are full of energy	1	2	3	4
You feel tense	1	2	3	4
Your problems seems to be pilling up	1	2	3	4
You feel you are in a hurry	1	2	3	4
You feel safe and protected	1	2	3	4
You have many worries	1	2	3	4
You are under pressure from other people	1	2	3	4
You feel discouraged	1	2	3	4
You enjoy yourself	1	2	3	4
You are afraid for the future	1	2	3	4
You feel you are doing things because you have to, not because you want to	1	2	3	4
You feel criticized or judged	1	2	3	4
You are light-hearted	1	2	3	4
You feel mentally exhausted	1	2	3	4
You have trouble relaxing	1	2	3	4
You feel loaded down with responsibility	1	2	3	4
You have enough time for yourself	1	2	3	4
You feel under pressure from deadlines	1	2	3	4

0=never; 1=almost never; 2=sometimes; 3=fairly often; 4=often.

Table S2. Epworth Slepiness Scale (ESS)

Situation	Chance of dozing
1. Sitting and reading	
2. Watching TV	
3. Sitting, inactive in a public place	
4. As a passenger in a car for an hour without break	
5. Lying down to rest in the afternoon when circumstances permit	
6. Sitting and talking to someone	
7. Sitting quietly after lunch without alcohol	
8. In a car, while stopped for a few minutes in the traffic	

0=would never dozing; 1=slight chance of dozing; 2=moderate chance of dozing; 3=high chance of dozing.

Table S3. Original Applied questionnaire.

1. Have you been diagnosed with COVID-19 infection?

a. Symptomatic disease		b. Asymptomatic disease
**Fever**	**Cough**	
**Shortness of breath**	**Myalgia**	
**chills**	**Heache**	
**Sore throat**	**New loss of taste or smell**	
**Nausea**	**Vomiting**	
**Diarhea**	**Fatigue**	

2. Have your family members been diagnosed with SARS-COV-2 infection?

a. Did you lived isolated from the rest of the family members?

3. Have you been working from home during pandemics?

a. Yes

b. No

c. No, I do not work anymore

3a. Do you consider you developed specific symptoms of depression or anxiety in the last 3 months?

3b. Being diagnosed with OSA, have you felt anxiety regarding SARS-COV-2 infection?

4. Has your sleep schedule modified in the last 3 months?

4a. How would you evaluate your sleep quality during pandemics?

a. Difficulties in getting asleep	e. Excessive daytime sleepiness
b. Difficulties in maintaining sleep	f. Hyperactive in the evening
c. Early wake-up	g. Unrefreshing sleep
d. Low or no tolerance of the CPAP	h. Agitation

4b. Do you use one of the following in order to improve your sleep quality?

a. Alcohol	b. Melatonin
c. Cannabis	d. Other

5. Has your weight modified in the last 3 months?

5a. You gained weight? If yes, how much kilograms?

5b. You lost weight? If yes, how much kilograms?

**6.**	**Do you consider that, being diagnosed with OSA, you have an increased risk for contacting COVID-19?**		
	6a. Did you search for information about COVID-19?<br>6b. Where did you get your information about COVID-19?		
**7.**	**If you consider your sleep routine changed, how did this influence you emotionally? (single or multiple answer)**		
a.	Fear for my health	b.	Worry for my family/friends
c.	Fear for my financial status	d.	Changing the sleep routine
e.	Loneliness	f.	Nightmares (before/after pandemics)
